# Correlation between dietary acid–base load and chronic kidney disease patients with type 2 diabetes mellitus

**DOI:** 10.3389/fnut.2025.1581009

**Published:** 2025-05-22

**Authors:** Hui Huang, Qian Wang, Ruimin Zhang, Fang Liu, Yue Niu, Yayong Luo, Shuang Li, Tao Li, Zhengchun Tang, Xiaolong Wang, Jian Yang, Yong Wang, Li Zhang, Sha Luo, Weiguang Zhang, Ying Zheng, Weizhu Deng, Guangyan Cai, Xiangmei Chen, Zheyi Dong

**Affiliations:** ^1^Chengdu University of Traditional Chinese Medicine, Chengdu, China; ^2^State Key Laboratory of Kidney Diseases, Beijing Key Laboratory of Medical Devices and Integrated Traditional Chinese and Western Drug Development for Severe Kidney Diseases, Beijing Key Laboratory of Digital Intelligent TCM for the Preventionand Treatment of Pan-vascular Diseases, Key Disciplines of National Administration of Traditional Chinese Medicine(zyyzdxk-2023310), Department of Nephrology, First Medical Center of Chinese PLA General Hospital, National Clinical Research Center for Kidney Diseases, Beijing, China; ^3^School of Clinical Medicine, Guangdong Pharmaceutical University, Guangzhou, China; ^4^Henan University of Traditional Chinese Medicine, Zhengzhou, China

**Keywords:** chronic kidney disease, type 2 diabetes mellitus, dietary acid load, potential renal acid load, net endogenous acid

## Abstract

**Objective:**

This study aimed to investigate the effect of dietary acid-base load on patients with chronic kidney disease (CKD) and type 2 diabetes mellitus (T2DM).

**Design:**

A total of 300 patients with CKD were enrolled and divided into three groups according to DAL tertiles. Dietary intake was assessed using a 24-h dietary recall, and diet-based acidity was assessed using net endogenous acid production (NEAP), potential renal acid load (PRAL), and dietary acid load (DAL). Multivariable logistic regression models were used to determine the association between diet-based acid load scores and CKD and T2DM.

**Results:**

Within the food category groupings, the DAL score was positively associated with poultry and eggs and negatively associated with fruits and vegetables. Regarding energy and macronutrients, the DAL score was positively correlated with the intake of protein, animal protein, monounsaturated fatty acids, saturated fatty acids, and fat, while it was negatively correlated with the intake of carbohydrates, plant protein, and dietary fiber. In terms of micronutrients, DAL scores were positively associated with phosphorus intake and negatively associated with potassium, magnesium, and copper intake. After adjusting for age, BMI, energy, and eGFR, and stratifying by sex, logistic regression analysis showed that DAL level (OR = 6.47, 95% CI 1.19–35.18, *p* = 0.031) was a related factor for CKD and T2DM in females.

**Conclusion:**

DAL score is a related factor for patients with T2DM and CKD.

## 1 Introduction

Chronic kidney disease (CKD) is a significant global public health concern due to its high morbidity, poor prognosis, and high mortality. In 2024, there were approximately 850 million patients with CKD worldwide, representing a global prevalence of about 9.5% (1). The prevalence of CKD in China is 8.2% (2). CKD is characterized by structural and functional changes in the kidneys lasting more than 3 months (3). Clinically, it involves multiple systems, including the respiratory, circulatory, skeletal, and endocrine systems, and eventually progresses to end-stage renal disease (ESRD). Diabetes and hypertension are the leading contributors to the increased CKD burden. The rising prevalence of diabetes has led to a steady increase in the prevalence of CKD combined with type 2 diabetes mellitus (T2DM). Patients with both CKD and T2DM are at greater risk compared to patients with CKD alone. Early intervention can delay CKD progression and renal failure. Additionally, it has been suggested that diet is an important etiologic and prognostic factor of CKD.

The acid–base status of the body can be influenced by dietary intake, depending on the balance between the intake of acid and alkaline-inducing foods. Diet significantly affects the risk of CKD progression (4). Dietary modifications that reduce acid load may improve prognosis in CKD and T2DM.Potential renal acid load (PRAL), net endogenous acid production (NEAP), and dietary acid load DAL are common metrics used to assess dietary acid–base load (5). Higher PRAL and NEAP scores indicate a greater acidogenic potential of the food. A diet with a high acid load can lead to low-grade metabolic acidosis, which can result in insulin resistance (6), T2DM (7) and metabolic syndrome (7). Furthermore, there are sex-related differences in the prevalence of T2DM (8). Taking sex-related differences into account in the recognition, development, presentation, diagnosis, treatment, and prevention of T2DM can facilitate the development of more personalized diabetes care strategies in the future.

This study aimed to evaluate the association between dietary acid–base load in patients with CKD and T2DM using PRAL, NEAP, and DAL scores, taking into account sex-related differences.

## 2 Materials and methods

### 2.1 Study population

This cross-sectional study, conducted between March 2022 and October 2023, involved adult patients with CKD hospitalized at the Department of Nephrology of the First Medical Center of the Chinese People’s Liberation Army General Hospital. The inclusion criteria comprised: (1) diagnosis of CKD according to the 2024 KDIGO Clinical Practice Guidelines (3), (2) age ≥ 18 years. The exclusion criteria were as follows: (1) history of severe infection within the past month; (2) acute and severe diseases in the past 6 months; (3) malignant tumors; (4) pregnancy or lactation; (5) incomplete medical history or clinical examination results; and (6) incomplete dietary intake data or over-reporting (> 4,000 kcal) and under-reporting of energy intake (< 600 kcal). Ultimately, 300 non-dialysis patients with CKD were included in the analysis.

### 2.2 Dietary intake

Dietary intake was assessed through face-to-face interviews using 24-h dietary recall within 48 h of patient admission to the hospital. During the 24-h dietary review, the researchers directly queried the patients about their food consumption on the preceding day, detailing the variety and quantities of food aided by food pictures or models. Nutrient intake was calculated according to the Chinese Dietary Guidelines (2022 edition) (9) and Chinese Dietary Reference Intakes (2013 edition) (10). Adjustment for food and nutrient intake was performed using the residual energy method (11).

### 2.3 Dietary acid–base load

Common indicators of dietary acid–base load include NEAP, PRAL, and DAL. NEAP and PRAL were calculated based on the dietary intake of proteins and minerals. DAL was calculated using dietary protein, phosphorus, potassium, calcium, magnesium, height, and weight.

PRAL (mmol/d) = 0.49 × protein (g/day) + 0.037 × phosphorus (mg/day) − 0.021 × potassium (mg/day) − 0.026 × magnesium (mg/day) − 0.013 × calcium (mg/day) (12);

NEAP (mEq/d) = 54.5 × [protein(g/day)/potassium intake (mEq/day)] − 10.2 (13);

DAL (mmol/d) = PRAL + (body surface area [m^2^] × 41[mEq/d]/1.73 m^2^) (14);

The body surface area was calculated as follows = 0.007184 × height (cm) ^ 0.725 × weight (kg)^ 0.425 (15, 16).

### 2.4 Clinical data collection

General information collected included name, sex, height, weight, age, and medical history, including present illness, nephropathy, hypertension, and diabetes. Clinical laboratory parameters measured included white blood cell count (WBC), hemoglobin, total protein, albumin, haptoglobin, prealbumin, urea nitrogen, serum creatinine, estimated glomerular filtration rate (eGFR), serum cystatin C, 24-h urinary protein, serum uric acid, total cholesterol, triglycerides, fasting blood glucose (FBG), serum calcium, potassium, phosphorus, high-density lipoprotein cholesterol (HDL-C) and low-density lipoprotein cholesterol (LDL-C).

### 2.5 Statistical analysis

Measurement data with a normal distribution were presented as mean ± standard deviation, while non-normally distributed data were expressed as medians with interquartile ranges. Differences between different DAL tertiles were compared, and measurement data with normal distribution and homogeneity of variance were compared between groups using a one-way analysis of variance test. A non-parametric test was used for comparison between groups if the homogeneity of variance was not satisfied. Count variables were expressed as frequencies and percentages and analyzed using chi-square or Fisher’s exact tests. Logistic regression analysis was used to analyze the relationship between the DAL score and CKD combined with T2DM after controlling for the confounding effects of age, body mass index (BMI), energy intake, and eGFR. Statistical analyses were performed using SPSS version 26.0 for Mac software (SPSS Inc., Chicago, IL, United States). Statistical significance was set at p (or P-trend) < 0.05.

## 3 Results

### 3.1 Screening of the selected cases

**Figure 1:**
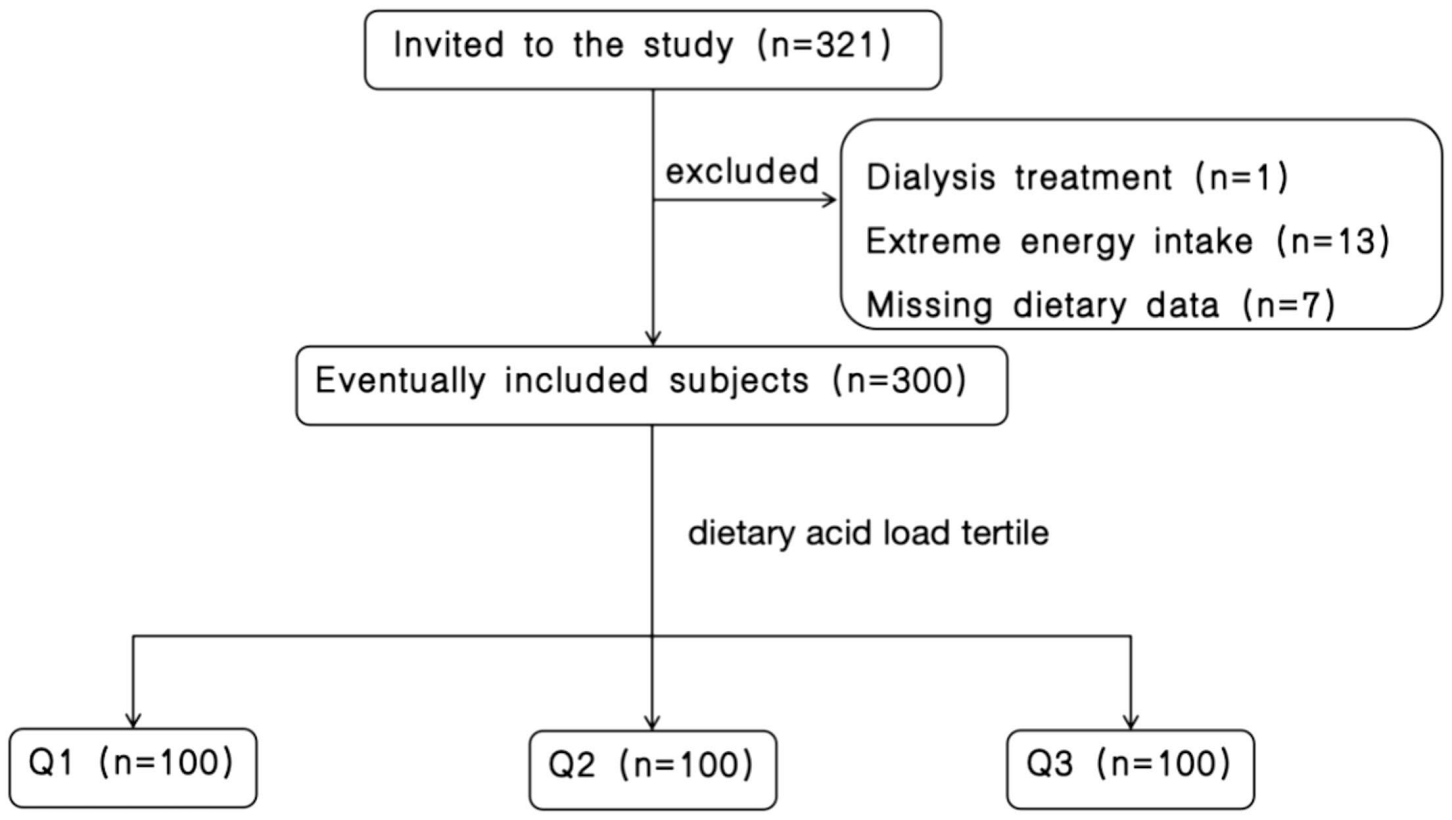
Flow chart of participant screening.

A total of 321 patients with CKD hospitalized at the Department of Nephrology of the First Medical Center of the Chinese People’s Liberation Army General Hospital between March 2022 and October 2023 were initially selected. After excluding seven patients due to incomplete dietary information, 13 patients with extreme energy intake (< 600 kcal/day or > 4,000 kcal/day), and one patient undergoing dialysis, 300 participants remained eligible for inclusion. Figure 1 provides a visual representation of the participant screening process.

### 3.2 General information on the study population

This study enrolled a total of 300 patients with CKD; approximately 63.7% were male. PRAL, NEAP, DAL, and BMI were significantly different in DAL tertiles, with an increasing trend (*p* < 0.001). Out of the total, 148 (49.3%) had T2DM, and significant differences appeared primarily between Q3 and Q1/Q2. No significant differences were observed in age, CKD course, or hypertension between the different DAL categories. Table 1 presents the characteristics of the 300 patients. Patients with T2DM were analyzed separately, Supplementary Table 1 shows baseline characteristics of patients combine CKD with T2DM by categories of DAL median.

**Table 1:** Baseline characteristics of study participants by categories of DAL.

Characteristics	*N* = 300	Q1 (*N* = 100)	Q2 (*N* = 100)	Q3 (*N* = 100)	*p*-value	*p*-trend
PRAL (mEq/day)	14.31 (5.64, 21.05)	0.35(−6.31, 5.73)	14.71 (10.57, 17.53)	23.93 (19.85, 28.75)	<0.001	<0.001
NEAP (mEq/day)	66.41 (51.88, 81.17)	44.62 (36.40, 53.26)	67.30 (59.70, 76.11)	83.88 (76.84, 101.30)	<0.001	<0.001
DAL (mEq/day)	55.7 (47.4, 64.6)	42.76 (34.76, 47.61)	55.70 (52.78, 58.87)	68.17 (64.57, 74.01)	<0.001	<0.001
Age (years)	54 (41, 61)	54 (43.25, 61)	54.5 (40, 62.75)	53.5 (42, 60)	0.801	0.509
Sex						
Male, n (%)	191 (63.7%)	52 (53%)	52 (53%)	87 (87%)	<0.001	–
Female, n (%)	109 (36.3%)	48 (48%)	48 (48%)	13 (13%)		
BMI (kg/m^2^)	25.01 ± 3.69	24.3 ± 3.66	24.17 ± 3.56	26.57 ± 3.36	<0.001	<0.001
CKD course (months)	24.5 (11.25, 84)	22.5 (10.25, 59.75)	32.5 (12, 106.25)	25 (12, 96)	<0.001	0.113
T2DM, n (%)	148 (49.3%)	45 (45%)	42 (45%)	61 (61%)	0.015	–
Hypertension, n (%)	226 (75.3%)	71 (71%)	74 (74%)	81 (81%)	0.242	–

PRAL, potential renal acid load; NEAP, net endogenous acid production; DAL, dietary acid load; BMI, body mass index; CKD, chronic kidney disease; T2DM, Type 2 Diabetes Mellitus.

### 3.3 Comparison of clinical characteristics of study participants

Blood urea nitrogen (BUN), cystatin C, uric acid, and FBG levels were significantly different in the DAL tertiles, with an increasing trend (*p* < 0.05). The participants assigned to the highest DAL category had significantly higher Serum creatinine levels, and significant differences appeared primarily between Q3 and Q1/Q2 (*p* < 0.05). The participants assigned to the highest DAL category had significantly lower HDL-C levels. In HDL-C levels, significant differences were observed particularly between the highest tertile and lower tertiles (*p* < 0.05).There were no significant differences in WBC count, hemoglobin, total protein, haptoglobin, prealbumin, eGFR, 24-h urinary protein, total cholesterol, triglyceride, serum calcium, serum potassium, serum phosphorus, or LDL-C levels among the different DAL categories. Table 2 presents a comparison of the clinical characteristics of the participants based on the DAL category.

**Table 2:** Comparison of clinical characteristics of study participants by categories of DAL.

Characteristics	*N* = 300	Q1 (*N* = 100)	Q2 (*N* = 100)	Q3 (*N* = 100)	*p*-value	*p*-trend
WBC (×10^9/L)	6.61 (5.21, 8.00)	6.74 (4.93, 8.37)	6.56 (5.32, 8.03)	6.46 (5.67, 7.74)	0.978	0.969
Hemoglobin (g/L)	118.5 (102.25, 131)	116 (100, 130)	116.82 (102, 128.75)	122 (106.5, 137.5)	0.094	0.068
Total protein (g/L)	58.85 (51.33, 64.8)	58.45 (49.25, 64.98)	58.25 (51.33, 63.38)	59.4 (52.7, 65.8)	0.619	0.781
Haptoglobin (mg/dl)	134.88 (85.58, 162)	134.88 (92.53, 165.75)	125.5 (70.2, 157.75)	133.94 (92.85, 164.5)	0.256	0.87
Prealbumin (mg/dl)	29.84 (24.43, 33)	28.5 (23.8, 32.58)	29.84 (24.7, 32.4)	30 (25.03, 35.43)	0.172	0.063
BUN (mmol/L)	8.11 (5.77, 11.36)	7.37 (5.38, 10.54)	8.05 (5.48, 11.72)	9.33 (6.41, 11.56)	0.028	0.008
Serum creatinine (umol/L)	116.25 (81.25, 188.23)	109 (77.48, 173.18)	99.9 (77.23, 157.98)	147.85 (88.73, 221.85)	0.023	0.018
eGFR (ml/min/1.73)	55.70 (30.66, 88.08)	60.49 (36.64, 90.55)	61.51 (32.81, 89.34)	42.77 (27.92, 82.1)	0.151	0.086
Cystatin C (mg/L)	1.81 (1.21, 2.00)	1.65 (1.11, 1.87)	1.81 (1.2, 2.00)	1.81 (1.36, 2.22)	0.102	0.035
24-h urinary protein (g/24 h)	2.03 (0.63, 4.56)	1.48 (0.51, 4.56)	2.35 (0.87, 4.24)	2.14 (0.82, 4.68)	0.301	0.128
Uric acid (umol/L)	375.65 (303.98, 455.8)	361.4 (274.83, 420.45)	373.9 (306.45, 438.48)	389.55 (323.58, 467.6)	0.017	0.004
Total cholesterol (mmol/L)	4.52 (3.84, 5.44)	4.56 (3.82, 5.49)	4.49 (3.86, 5.47)	4.55 (3.86, 5.37)	0.94	0.727
Triglyceride (mmol/L)	1.8 (1.22, 2.56)	1.75 (1.21, 2.44)	1.59 (1.14, 2.38)	1.94 (1.45, 2.76)	0.011	0.065
FBG (mmol/L)	4.79 (4.29, 5.64)	4.59 (4.16, 5.25)	4.85 (4.38, 5.50)	4.87 (4.37, 6.32)	0.098	0.041
Serum calcium (mmol/L)	2.19 (2.06, 2.27)	2.21 (2.06, 2.28)	2.19 (2.07, 2.27)	2.17 (2.07, 2.26)	0.568	0.292
Serum potassium (mmol/L)	3.90 (3.62, 4.26)	3.91 (3.71, 4.31)	3.87 (3.61, 4.22)	3.90 (3.55, 4.26)	0.493	0.242
Serum phosphorus (mmol/L)	1.27 (1.13, 1.42)	1.27 (1.12, 1.41)	1.28 (1.14, 1.43)	1.26 (1.12, 1.43)	0.767	0.504
HDL-C (mmol/L)	1.1 (0.9, 1.32)	1.13 (0.92, 1.5)	1.15 (0.95, 1.43)	1.03 (0.86, 1.17)	0.001	0.003
LDL-C (mmol/L)	2.68 (2.08, 3.50)	2.73 (2.09, 3.62)	2.65 (2.12, 3.34)	2.69 (2, 3.50)	0.925	0.703

WBC, white blood cell; BUN, blood urea nitrogen; eGFR, estimated glomerular filtration rate; FBG, fasting blood glucose; HDL-C, high-density lipoprotein cholesterol; LDL-C, low-density lipoprotein cholesterol.

### 3.4 Distribution of dietary intake in the study population

Among the food groups, DAL was positively associated with meat and eggs (*p* < 0.001) but negatively associated with fruits and vegetables (*p* < 0.001). Regarding energy and macronutrients, DAL was positively associated with protein, animal protein, monounsaturated fatty acids (MUFA), saturated fatty acids, and fat intake (*p* < 0.05) while displaying a negative association with carbohydrate, plant protein, and fiber intake (*p* < 0.001). No significant association was found in polyunsaturated fatty acid (PUFA) intakes across the DAL tertiles.

Concerning dietary micronutrient intake, DAL was negatively associated with sodium, potassium, calcium, magnesium, and iron while being positively associated with phosphorus and zinc intake. Specifically, DAL was negatively associated with potassium, magnesium, and copper intakes (*p* < 0.05), but it was positively associated with phosphorus intake (*p* < 0.05). Table 3 presents the dietary intakes of the participants in the DAL tertiles.

**Table 3:** Food groups, macronutrient, and micronutrient intake of study participants by categories of DAL.

Variables	*N* = 300	Q1 (*N* = 100)	Q2 (*N* = 100)	Q3 (*N* = 100)	*p*-value	*p-*trend
Food groups						
Grain (g/day)	327.5 (223.85, 487.15)	360 (230, 493.08)	303.55 (215.35, 445.3)	323 (222.2, 504.58)	0.297	0.715
Vegetables (g/day)	297.9 (175.75, 435.9)	398.9 (241.15, 586.5)	280.55 (188, 387.5)	241.45 (132.15, 328.75)	<0.001	<0.001
Fruits (g/day)	100 (0, 207.83)	200 (40.55, 400)	105 (0, 200)	0 (0, 150)	<0.001	<0.001
meat(g/day)	60 (14.25, 140)	37.5 (0, 95.48)	35 (10, 99.65)	130.45 (50, 199.7)	<0.001	<0.001
Eggs (g/day)	60 (0,60)	39.5 (0, 60)	60 (0, 85.15)	60 (0, 90)	0.005	0.002
Dairy products (ml/day) energy and Macronutrients	0 (0, 250)	0 (0, 200)	0 (0, 250)	0 (0, 250)	0.947	0.767
Energy (kcal/day)	1320.30 (1021.89, 1653.56)	1371.06 (1027.04, 1659.27)	1180.68 (954.85, 1524.49)	1413.85 (1142.26, 1943.87)	0.001	0.116
Carbohydrates (g/day)	213.36 (186.96, 238.91)	222.57 (203.37, 252.30)	213.08 (192.44, 238.44)	194.51 (163.95, 229.27)	<0.001	<0.001
Protein (g/day)	59.50 (53.07, 68.11)	54.56 (49.86, 59.37)	59.82 (54.40, 66.40)	68.02 (59.08, 81.52)	<0.001	<0.001
Plant protein (g/day)	31.50 (26.01, 36.55)	32.87 (28.67, 37.88)	30.48 (26.49, 37.01)	31.15 (22.87, 36.15)	0.099	0.032
Animal protein (g/day)	28.95 (18.85, 39.51)	22.26 (14.94, 30.26)	28.45 (19.83, 39.42)	37.91 (26.59, 53.40)	<0.001	<0.001
Fiber (g/day)	8.01 (5.44, 10.95)	10.98 (8.10, 13.97)	7.88 (6.14, 9.78)	5.67 (3.60, 8.16)	<0.001	<0.001
MUFA (mg/day)	11.57 (8.13, 16.39)	10.13 (6.06, 14.30)	11.49 (8.27, 15.24)	13.72 (9.37, 18.62)	0.002	<0.001
PUFA (mg/day)	3.93 (2.54, 5.20)	3.67 (2.28, 4.86)	4.09 (2.71, 5.31)	4.19 (2.73, 6.02)	0.155	0.055
Saturated fat (mg/day)	12.93 (8.54, 16.64)	11.11 (6.83, 14.72)	13.04 (8.44, 16.14)	14.76 (11.33, 19.99)	<0.001	<0.001
Fat (g/day)	39.93 (29.95, 48.09)	37.56 (27.89, 46.15)	40.19 (29.97, 47.48)	44.13 (32.59, 54.05)	0.022	0.006
Micronutrients						
Sodium (mg/day)	1018.09 (730.44, 1708.44)	1031.89 (741.23, 1721.05)	977.42 (734.15, 1505.81)	1048.50 (722.85, 1888.30)	0.709	0.522
Potassium (mg/day)	1721.50 (1414.87, 2138.99)	2156.01 (1776.72, 2460.72)	1612.03 (1404.79, 1939.10)	1512.17 (1251.83, 1823.32)	<0.001	<0.001
Calcium (mg/day)	369.18 (248.04, 548.83)	418.711 (258.43, 639.90)	348.19 (251.14, 534.64)	355.61 (203.41, 521.60)	0.017	0.005
Magnesium (mg/day)	261.61 (220.58, 303.10)	293.54 (250.17, 357.17)	252.26 (221.97, 284.68)	230.45 (207.07, 275.50)	<0.001	<0.001
Phosphorus (mg/day)	854.93 (754.41, 1004.59)	831.30 (732.08, 986.92)	848.02 (761.21, 932.82)	919.70 (787.47, 1082.90)	0.017	0.018
Iron (mg/day)	14.93 (11.84, 17.89)	15.22 (12.37, 18.72)	14.62 (11.98, 16.98)	14.59 (11.26, 17.50)	0.277	0.141
Copper (mg/day)	1.14 (0.87, 1.47)	1.33 (1.02, 1.74)	1.14 (0.92, 1.35)	0.98 (0.77, 1.22)	<0.001	<0.001
Zinc (mg/day)	7.3 (6.09, 8.81)	7.42 (6.13, 9.06)	7.02 (6.04, 8.01)	7.64 (6.02, 10.06)	0.161	0.511

MUFA, mono-unsaturated fatty acids; PUFA, polyunsaturated fatty acids.

### 3.5 Logistic regression analysis was used to analyze the relationship between dietary acid–base load and CKD combined with T2DM

Regarding the association between dietary acid–base load and CKD combined with T2DM, odds ratios (ORs) and 95% confidence intervals (CI) for the association between dietary acid and base are shown in Table 4. In the crude model, the DAL score was significantly associated with higher odds in CKD combined with T2DM. Specifically, the risk of CKD combined with T2DM in the highest tertile of DAL was 91% greater than that in the lowest tertile (OR = 1.91, 95%CI 1.09–3.36, *p* = 0.024). After adjusting for age, BMI, the DAL score was significantly associated with higher odds of patients with CKD and T2DM. The risk of CKD combined with T2DM in the highest tertile of DAL was 2.07 times greater than that in the lowest tertile (OR = 2.07, 95%CI 1.06–4.03, *p* = 0.033). Similarly, In Model 2, the odds of CKD combined with T2DM in the highest DAL tertile were 2.06 times greater than those in the lowest tertile (OR = 2.06, 95%CI 1.05–4.01, *p* = 0.035). Subsequently, in Model 3, when further adjusted for eGFR, no significant association was observed between DAL in CKD combined with T2DM. No significant association was observed between PRAL or NEAP levels in CKD combined with T2DM.

**Table 4:** Logistic regression analysis models for the association between CKD combined with T2DM and tertiles of PRAL, NEAP, and DAL.

Variables			PRAL			NEAP			DAL		
			Q1	Q2	Q3	Q1	Q2	Q3	Q1	Q2	Q3
T2DM	Crude	OR	1	0.79	1.17	1	0.82	1.27	1	0.89	1.91
		95%CI		0.45–1.37	0.67–2.05		0.47–1.43	0.73–2.22		0.51–1.55	1.09–3.36
		*p*		0.396	0.571		0.479	0.396		0.669	0.024
	Model 1	OR	1	0.6	1.32	1	0.68	1.34	1	0.89	2.07
		95%CI		0.31–1.15	0.69–2.53		0.35–1.31	0.71–2.55		0.46–1.72	1.06–4.03
		*p*		0.124	0.404		0.251	0.371		0.736	0.033
	Model 2	OR	1	0.601	1.317	1	0.69	1.33	1	0.90	2.06
		95%CI		0.31–1.17	0.69–2.53		0.36–1.33	0.70–2.54		0.47–1.75	1.05–4.01
		*p*		0.136	0.406		0.265	0.381		0.761	0.035
	Model 3	OR	1	0.66	1.21	1	0.72	1.26	1	0.93	1.92
		95%CI		0.33–1.31	0.62–2.37		0.37–1.43	0.65–2.45		0.47–1.85	0.96–3.82
		*p*		0.23	0.572		0.354	0.492		0.845	0.064

Crude: unadjusted; Model 1: adjusted for age and body mass index; Model 2: adjusted for age, body mass index and energy intake; Model 3: adjusted for age, body mass index, energy intake and eGFR. DAL, dietary acid load; NEAP net endogenous acid production; PRAL, potential renal acid load.

### 3.6 Logistic regression analysis models for the association between CKD combined with T2DM and tertiles of PRAL, NEAP, and DAL categorized by sex

The results of crude and multivariable adjusted ORs and 95% CI for the association between CKD combined with T2DM and tertiles of PRAL, NEAP, and DAL scores stratified by sex are presented in Table 5. In males, no significant association was observed between any indices of dietary acid–base load and CKD combined with T2DM. However, in females, higher DAL scores were significantly associated with higher odds of CKD combined with T2DM. In Models 1, 2, and 3, the risk of CKD combined with T2DM in the highest DAL tertile was 6.61, 6.57, and 6.47 times higher, respectively, than that in the lowest DAL tertile. 1. No significant modifications were observed between PRAL, NEAP, and DAL and CKD combined with T2DM (*p* > 0.05; Supplementary Table 2).

**Table 5:** Logistic regression analysis models for the association between CKD combined with T2DM and tertiles of PRAL, NEAP, and DAL categorized by sex.

Variables			PRAL			NEAP			DAL		
			Q1	Q2	Q3	Q1	Q2	Q3	Q1	Q2	Q3
Male	Crude	OR	1	0.59	0.95	1	0.65	0.94	1	0.58	1.07
		95%CI		0.28–1.23	0.47–1.91		0.31–1.36	0.47–1.89		0.27–1.26	0.53–2.18
		*p*		0.159	0.88		0.252	0.86		0.169	0.843
	Model 1	OR	1	0.49	1.07	1	0.61	1.01	1	0.65	1.21
		95%CI		0.21–1.13	0.49–2.36		0.26–1.43	0.46–2.19		0.26–1.59	0.55–2.68
		*p*		0.095	0.863		0.254	0.983		0.342	0.632
	Model 2	OR	1	0.45	1.07	1	0.6	1.01	1	0.63	1.20
		95%CI		0.19–1.07	0.48–2.35		0.25–1.41	0.46–2.19		0.25–1.55	0.54–2.65
		*p*	1	0.072	0.873		0.242	0.987		0.31	0.654
	Model 3	OR		0.51	0.96	1	0.62	0.93	1	0.65	1.16
		95%CI		0.21–1.25	0.42–2.20		0.26–1.50	0.41–2.10		0.26–1.65	0.51–2.65
		*p*		0.14	0.931		0.287	0.855		0.361	0.722
Female	Crude	OR	1	1.4	1.23	1	1.32	1.59	1	1.48	2.31
		95%CI		0.55–3.56	0.42–3.56		0.52–3.36	0.555–4.564		0.62–3.52	0.65–8.16
		*p*		0.477	0.706		0.564	0.388		0.38	0.194
	Model 1	OR	1	0.97	1.62	1	0.95	1.9	1	1.81	6.61
		95%CI		0.31–3.06	0.45–5.85		0.30–2.99	0.53–6.80		0.59–5.53	1.25–35.09
		*p*		0.959	0.463		0.933	0.326		0.3	0.027
	Model 2	OR	1	0.99	1.61	1	0.96	1.87	1	1.81	6.57
		95%CI		0.31–3.18	0.45–5.81		0.30–3.03	0.52–6.74		0.59–5.56	1.21–35.70
		*p*	1	0.993	0.468		0.944	0.339		0.3	0.029
	Model 3	OR		1.03	1.64	1	1.01	1.96	1	1.87	6.47
		95%CI		0.32–3.36	0.46–5.92		0.32–3.24	0.54–7.11		0.60–5.81	1.19–35.18
		*p*		0.957	0.45		0.987	0.305		0.279	0.031

Crude: unadjusted; Model 1: adjusted for age and body mass index; Model 2: adjusted for age, body mass index and energy intake; Model 3: adjusted for age, body mass index, energy intake and eGFR. DAL, dietary acid load; NEAP, net endogenous acid production; PRAL, potential renal acid load.

## 4 Discussion

### 4.1 Association between dietary acid–base load and CKD with T2DM

Metabolic acidosis occurs when the kidneys do not fully excrete the acid load generated by metabolism. Metabolic acidosis is a common complication in patients with CKD. The potentially deleterious effects of metabolic acidosis in CKD include muscle wasting, bone demineralization, hyperkalemia, and accelerated disease progression (17). One study showed that metabolic acidosis caused proteinuria and decreased eGFR, associated with the intrarenal renin-angiotensin system activation (18). Elevated dietary acid intake can also provoke tubular toxicity, triggering the complement pathway and boosting renal medullary ammonia levels (19). Diet plays a crucial role in generating nonvolatile acid loads. In an elderly Spanish population study targeting metabolic syndrome, higher PRAL and NEAP scores were associated with decreased eGFR and increased urine albumin creatine ratio (UACR) after 1 year of follow-up (20). A meta-analysis also confirmed the association between dietary acid–base load and renal function, with a high dietary acid load leading to decreased renal function (21). After adjusting for multiple confounders, a higher dietary acid load was associated with a higher prevalence of CKD or impaired renal function, as indicated by low eGFR (22–24). A large body of evidence links dietary acid load to renal outcomes.

Recent research has focused on the relationship between diet-induced acid–base load, T2DM, and insulin resistance (6, 25–29). A pooled study of three prospective cohorts showed that a higher diet-dependent acid load (including NEAP, PRAL, and animal protein-to-potassium ratio) was associated with an increased risk of T2DM (25). Similarly, a prospective study in a middle-aged and elderly Korean population found that a higher PRAL score was associated with an increased risk of future insulin resistance (6). Additionally, a meta-analysis of 14 studies demonstrated that participants with the highest PRAL and NEAP scores had a 19 and 22% increased risk of diabetes mellitus (DM), respectively, compared to those in the lowest category (30). Similarly, the DAL score was positively associated with CKD and T2DM in our study. The mechanisms by which DAL specifically affects the progression of T2DM pathophysiology may be as follows. Firstly, high DAL results in low interstitial fluid PH. Insulin receptors are found on the plasma membrane, particularly in insulin-targeted cells. When insulin hooks onto its receptor, it does so on the side that faces the surrounding interstitial fluid. Therefore, alterations in the microenvironments of interstitial fluids, particularly pH levels, influence the insulin’s binding strength with its receptor (31, 32). Secondly, High DAL enhance adrenal cortex cortisol production (33). Chronically elevated cortisol levels can lead to insulin resistance (34). DAL modification could be a feasible intervention target. The Mediterranean diet is mainly a plant-based diet and belongs to a low diet-based acidity. The Mediterranean diet is recommended by the National Kidney Foundation’s Kidney Disease Outcomes Quality Initiative (35). The study has shown that the Mediterranean diet can reduce FBG, glycated hemoglobin and LDL-C (36). There is evidence that adhering to a Mediterranean diet lowers the likelihood of developing T2DM (37, 38).

### 4.2 Association between dietary factors and risks of CKD and T2DM

In our study, the food groups in the highest tertile of DAL scores were characterized by low consumption of vegetables and fruits but high consumption of meat and eggs. Food groups with high DAL scores are characterized by low consumption of vegetables and fruits and high consumption of dairy products, cereals, and eggs (39). Vegetables and fruits have excellent antioxidant properties that help eliminate free radicals and mitigate oxidative stress, thus protecting cells and structures from oxidative damage (40). A high intake of fruits and vegetables has long been associated with health benefits such as protecting against cancer, diabetes, neurodegenerative diseases, and cardiovascular diseases (41, 42). On the other hand, increased meat intake, particularly synthetic and processed meat, has been positively associated with proinflammatory substances (43). Animal proteins, rich in sulfur-containing amino acids such as methionine, homocysteine, and cysteine, contribute to the production of sulfate with an acidification effect during the metabolic process. This can lead to metabolic acidosis (44, 45).

In this study, in terms of energy and macronutrients, patients with CKD and T2DM in the highest DAL tertiles consumed more protein, including animal protein, as well as MUFA, saturated fatty acids, fat, and phosphorus. Conversely, their intake of carbohydrates, plant protein, dietary fiber, potassium, magnesium, and copper was lower. Some studies suggest that total and animal proteins can increase the risk of T2DM, while plant proteins can reduce the risk of T2DM (46, 47). Hence, it is essential to consider both the type and food source of dietary proteins for the prevention of diabetes.

Several studies have provided evidence supporting an association between dietary fatty acids, especially saturated fatty acids, and the development of insulin resistance and T2DM. However, recent cohort studies have shown no association between saturated fatty acids and the incidence of T2DM (48, 49). Conversely, several prospective studies have demonstrated an inverse association between total MUFA intake and the risk of T2DM (50, 51). There may be the following reasons the negative association between DAL and MUFA in this study. Firstly, Traditional Chinese cooking, such as stir frying, pan frying, and deep frying, can lead to isomerization of unsaturated fatty acids (UFA), which in turn produces harmful substances such as trans fatty acids (52, 53). Secondly, 24-h dietary review do not represent long-term dietary intake habits. Higher concentrations of marine-derived omega-3 PUFA biomarkers have been linked to greater risks of T2DM, coronary heart disease, and total mortality (54). Conversely, another study reported that higher levels of omega-3 PUFA in seafood sources were associated with a lower risk of CKD (55). Whether fatty acids are risk factors or protective factors for T2DM remains controversial, potentially influenced by differences in food sources and carbon chain lengths (48, 56).

### 4.3 Sex disparities in the dietary acid–base load and CKD with T2DM

Three distinct dietary acid–base load measures were employed in this study, each with its unique formula, limitations, and advantages. Consequently, utilizing these three indicators enhances the reliability of the results. In a cross-sectional study of 1945 Iranian adults, participants in the highest quartile of PRAL, NEAP, and DAL had 42, 48, and 44% higher ORs for metabolic syndrome compared to those in the lowest quartile, respectively. Subsequent analysis of the population by sex showed that higher PRAL, NEAP, and DAL scores were significantly associated with increased odds of metabolic syndrome in the male population but not in the female population (7). Notably, no previous study has investigated the association between these three dietary acid–base load measures (PRAL, NEAP, and DAL) in patients with CKD and T2DM while considering sex differences. Further analysis by sex showed that in women in Models 1, 2, and 3, the risk of CKD with T2DM in the highest DAL tertile was 6.61, 6.57, and 6.47 times higher than that in the lowest DAL tertile. However, no significant association was observed among males.

Evidence suggests that sex differences play an important role in the pathophysiology, epidemiology, treatment, and prognosis of metabolic syndrome and DM. There are sex-related differences in the prevalence of T2DM. Globally, the prevalence of DM is higher in men, but more women are affected by T2DM than men (57). The sex difference in DM prevalence is reversed according to the reproductive life stage: prepubertal males exhibit higher rates of DM, whereas postmenopausal and older women have more DM. Gestational diabetes mellitus (GDM) is the most important risk factor for the development of T2DM in women (58). Additionally, premature menopause is associated with an increased risk of T2DM (59–61). This reason is caused by hormonal differences in metabolic responses between genders. Estrogen protects against insulin resistance by activating the ERα pathway in insulin-sensitive tissues (62). Estrogen decreases in women after menopause. Low estrogen levels increase inflammation and enhances fat accumulation in the body. Excess fat produces excess triglycerides and free fatty acids, which in turn impair insulin signaling and *β*-cell regulation, leading to T2DM (63). Estrogen influences microbiota in the gut, the gut microbiota regulates glucose homeostasis (64).Endogenous estrogens play a protective role in preventing females from developing type 2 diabetes. Men have small fluctuations in hormone levels throughout life compared with women. Androgen is converted into estrogen through the process of aromatization. Men with an androgen/estrogen ratio imbalance are at metabolic risk.

Taking gender differences into account in the recognition, development, presentation, diagnosis, treatment, and prevention of T2DM will facilitate the development of more personalized diabetes care in the future.

### 4.4 Strengths and limitations

This study is the first to use three measures (PRAL, NEAP, and DAL) to assess the association between dietary acid–base load and the risk of combined CKD and T2DM while considering disparities in sex. However, we excluded all subjects with extreme values of total energy intake and adjusted the intake of food and nutrients according to the energy residual method to control for reporting bias.

Nonetheless, this study has some limitations. Firstly, the dietary data of the patients did not encompass the use of supplements. Secondly, this was a cross-sectional, single-center study, and it was not able to determine cause and effect. Longitudinal design or interventional studies are recommended to further verify causality. The diversity and representativeness of the samples may be limited. Future studies be validated in a broader population, including patients with different regions, ethnicities, and medical backgrounds. Thirdly, the 24-h dietary review was used to assess dietary intake, which may be subject to recall bias. It is recommended that future studies use more accurate dietary assessment methods (such as food diaries or multiple 24-h reviews). Fourthly, despite accounting for potential confounding elements, other potential confounders may still exist, such as physical activity levels, medication use, or socioeconomic status.

## 5 Conclusion

In conclusion, our study revealed a positive association between the DAL scores and CKD with T2DM, particularly among women. An alkaline - rich diet, which is abundant in fruits and vegetables and lower in excessive meat, might be a modifiable factor in reducing DAL. Additional studies are required to ascertain the specific foods’ acidogenic potential to establishing specific dietary recommendations.

## Data Availability

The original contributions presented in the study are included in the article/Supplementary material, further inquiries can be directed to the corresponding authors.
