# Supplementary material for: Correlation between dietary acid–base load and chronic kidney disease patients with type 2 diabetes mellitus
Source: Front Nutr. 2025 May 22;12:1581009. doi: 10.3389/fnut.2025.1581009 (PMC12137085; doi:10.3389/fnut.2025.1581009)
Supplement: Supplementary file 1 [file Table_1.docx]

Supplementary Material

# Supplementary Table 1

Supplementary Table 1:Baseline characteristics of patients combine CKD with T2DM by categories of DAL median.

| Characteristics | N=148 | T1（N=74） | T2（N=74） | P |
| --- | --- | --- | --- | --- |
| PRAL (mEq/day) | 15.89(5.66,21.33) | 5.67(-0.92,11.35) | 21.33(18.53,26.93) | ＜0.001 |
| NEAP (mEq/day) | 68.79(51.88,82.51) | 51.90(42.56,62.22) | 80.7(75.33,97.14) | ＜0.001 |
| DAL (mEq/day） | 57.83±13.17 | 47.11±7.33 | 68.56±7.88 | ＜0.001 |
| Age (years) | 59(52,64.75) | 60(55,66) | 58(50,64) | 0.069 |
| Male, n (%) | 112(75.7%) | 48(64.9%) | 64(86.6%) | 0.002 |
| BMI（kg/m²） | 25.81±3.14 | 24.83±3.17 | 26.8±2.79 | ＜0.001 |
| CKD course (months) | 29.5(12,99.75) | 25(10.75,70.25) | 36(13.5,120) | 0.186 |
| DM (months) | 120(24,204) | 120(45,192) | 120(24,210) | 0.991 |
| Hypertension，n（%） | 133(89.9%) | 65(87.8%) | 68(91.9%) | 0.414 |

PRAL, potential renal acid load; NEAP, net endogenous acid production; DAL, dietary acid load; BMI, body mass index; CKD, chronic kidney disease; T2DM, DM,Diabetes duration.

# Supplementary Table 2

Supplementary Table 2:Logistic regression analysis models for the association between combine CKD with T2DM and tertiles of PRAL, NEAP, and DAL categorized by eGFR.

| eGFR  (ml/min/1.73) | | PRAL | | | NEAP | | | DAL | | |
| --- | --- | --- | --- | --- | --- | --- | --- | --- | --- | --- |
|  |  | Q1 | Q2 | Q3 | Q1 | Q2 | Q3 | Q1 | Q2 | Q3 |
| ≥60 | OR | 1 | 0.66 | 0.98 | 1 | 0.7 | 0.99 | 1 | 1.01 | 1.31 |
|  | 95%CI |  | 0.23-1.89 | 0.35-2.76 |  | 0.25-1.98 | 0.36-2.73 |  | 0.37-2.81 | 0.46-3.71 |
|  | p |  | 0.442 | 0.968 |  | 0.497 | 0.984 |  | 0.978 | 0.614 |
| <60 | OR | 1 | 0.54 | 1.43 | 1 | 0.69 | 1.59 | 1 | 0.86 | 2.48 |
|  | 95%CI |  | 0.21-1.43 | 0.57-3.56 |  | 0.28-1.73 | 0.64-3.97 |  | 0.34-2.17 | 0.95-6.48 |
|  | p |  | 0.214 | 0.443 |  | 0.427 | 0.32 |  | 0.742 | 0.065 |

Adjusted for age, body mass inde, energy intake and eGFR.DAL, dietary acid load; NEAP net endogenous acid production; PRAL, potential renal acid load.
